# Single cell Raman spectroscopic profiles predict treatment responses in patients with *de novo* acute myeloid leukemia

**DOI:** 10.3389/fcell.2026.1767226

**Published:** 2026-04-28

**Authors:** Ming Zhang, Long Su, Wei Han, Fei Song, Yu Fu, Ming-Bo Chi, Xing Chen, Yi-Hui Wu, Su-Jun Gao

**Affiliations:** 1 Department of Hematology, The First Hospital of Jilin University, Changchun, China; 2 Key Laboratory of Hematology Precision Medicine of Jilin Province, The First Hospital of Jilin University, Changchun, China; 3 Changchun Institute of Optics, Fine Mechanics and Physics, Chinese Academy of Sciences, Changchun, China

**Keywords:** acute myeloid leukemia, prediction, Raman spectral analysis, remission, therapeutic respsonse

## Abstract

**Introduction:**

Leukemia is a clonal malignant proliferative disease originating from hematopoietic stem cells. Although its treatment strategy has gradually developed from traditional chemotherapy to a multimodal treatment system including novel targeted therapy and immunotherapy, primary drug resistance in particular remains the core clinical problem leading to poor patient prognosis. This clinical dilemma indicates that the traditional genotyping system based on genomics has not been able to fully resolve the molecular heterogeneity of acute myeloid leukemia (AML), and it is urgent to establish a precise stratified model that can dynamically reflect the functional status of tumor cells in the initial stage of treatment.

**Methods:**

In this study, Raman spectroscopy (RS) combined with machine learning algorithm was used to construct a metabolic prognosis prediction model for AML chemotherapy response. Bone marrow single cell Raman spectroscopy data of newly diagnosed AML patients were collected, and the molecular fingerprint was analyzed by principal component analysis linear discriminant analysis (PCA-LDA) and multivariate curve resolute alternating least square method (MCR-ALS).

**Results:**

The results showed that the PCALDA model achieved complete remission or non-remission (CR/NR) classification through 24 principal components (cumulative variance contribution of 90.1%), the accuracy of external validation was 94.8% (sensitivity 97.9%, specificity 92.0%), and the AUC reached 96.27%. Protein, lipid, nucleic acid and mixed components were decomposed by MCR-ALS, and lipid and nucleic acid metabolic pathways were enriched in NR group (P < 0.001).

**Discussion:**

Studies have shown that RS single-cell metabolic fingerprint can decode the metabolic reprogramming features associated with chemotherapy resistance in AML, providing a new marker-free and highly sensitive tool for real-time prognostic stratification and targeted intervention.

## Introduction

Acute myeloid leukemia (AML) is a malignant hematologic disorder characterized by the clonal proliferation and impaired differentiation of myeloid precursor cells (DiNardo et al., 2023). The current standard treatment involves initial induction chemotherapy followed by post-remission consolidation therapy (Yeung and Radich, 2017; Santoro et al., 2024; Döhner et al., 2022). The primary goal of AML treatment is to achieve complete remission (CR) through initial therapy, with the first course aiming to induce CR and improve long-term survival (Döhner et al., 2017). However, approximately 10%–40% of newly diagnosed patients fail to achieve CR after initial induction therapy, reflecting significant heterogeneity in therapeutic response (Thol et al., 2024; Newell and Cook, 2021). Those patients show a poor prognosis, characterized by lower response rates to conventional salvage chemotherapy and shorter overall survival compared to patients who are sensitive to initial therapy (Qi et al., 2023; Wheatley et al., 1999). Therefore, a deeper understanding of disease biology and optimization of treatment protocols are crucial for improving patients’ outcomes. An increasing body of research indicates a significant correlation between AML prognosis and metabolic status (Shi et al., 2024; Chen et al., 2021). As our insight into the cellular events expands, the ability to analyze the biochemical composition of single cells including small molecules, proteomic and genomic profiles, and epigenetic features has become increasingly important (Grimwade et al., 2016). Understanding the heterogeneity of refractory AML and the distinct cellular characteristics is essential for deciphering the mechanisms underlying therapy resistance. Metabolic heterogeneity is a key determinant of AML progression and treatment failure; however, there are limited studies to investigate the impact of metabolic perturbations on fine-grained risk stratification and personalized management decisions for AML patients (Pino et al., 2024). A critical reason underlying this gap is the lack of effective detection methods.

Raman spectroscopy (RS), a non-invasive optical analysis technique, acquires molecular fingerprints by detecting specific chemical bond vibration modes (Gaba et al., 2022; Cutshaw et al., 2023). This technology enables rapid detection and molecular conformation analysis of biological samples (including amino acids, phospholipids, carbohydrates and carboxylic acids, among others), demonstrating significant advantages in elucidating dynamic compositional and structural changes in biomolecules (Draux et al., 2010; Aguiar et al., 2022). Leveraging its molecular specificity and high spatial resolution, RS provides a unique perspective for exploring the mechanisms of biological activity and holds substantial promise for disease diagnosis, therapeutic efficacy monitoring, and prognosis evaluation (Gaba et al., 2022; Zhang et al., 2023). Recently, RS has achieved notable progress in AML precise diagnosis and molecular subtyping due to its label-free detection capability, single-cell resolution, and molecular fingerprinting characteristic (Eberhardt et al., 2015; Bai et al., 2020; Liang et al., 2022). Liang et al. employed an orthogonal partial least squares discriminant analysis model to extract features from bone marrow supernatant spectra, successfully distinguishing AML from acute lymphoblastic leukemia (ALL) (Liang et al., 2022). Regarding subtype identification, Vanna and co-workers developed a spectral analysis algorithm based on single-step global clustering, enabling semi-quantitative characterization of hyperspectral data from bone marrow cells (Vanna et al., 2024). This approach offers a more objective, automated, and accurate method for leukemia subtype classification and diagnosis, overcoming the subjectivity, time consumption, and observer variability inherent in conventional morphological assessment. Ye et al. integrated surface-enhanced Raman spectroscopy (SERS) with proton nuclear magnetic resonance (^1^H-NMR) metabolomics, revealing significant differences in metabolic profiles between AML subtypes and prognostic risk subgroups (Ye et al., 2022). Specific SERS bands at 1328 cm^−1^ and 741 cm^−1^, both attributed to nucleic acid signals, were identified as potential AML subtype markers. In terms of therapeutic resistance, Mojidra and colleagues utilized Raman to distinguish imatinib-sensitive from drug-resistant cells, achieving an overall accuracy of 93.9% (Mojidra et al., 2021). Our previous study demonstrated that AML with FLT3-ITD mutations exhibited distinct spectral features in nucleic acids and proteins (Wang et al., 2023). Additionally, Raman spectroscopy can capture distinctive spectral phenotypes associated with specific genetic mutations (e.g., *NPM1*) of AML subtypes. This capability underscores its potential clinical utility for phenotypic subtyping (Li et al., 2023). Nevertheless, most existing studies focus on morphological characteristics and genetic alterations, while the mechanistic links between metabolic dysregulation and treatment situation remain poorly explored. Early identification of therapeutic determinants and development of personalized treatment strategies remain a crucial clinical challenge.

In this study, we innovatively constructed a prediction model linking RS-based fingerprint metabolic profiles to AML treatment response. Using machine learning algorithms PCA-LDA (Kongklad et al., 2022; Jeng et al., 2019) and MCR-ALS (Castro et al., 2021; Matveeva et al., 2022), the correlation between the biochemical heterogeneity of leukemia cells and the clinical treatment response of newly diagnosed AML patients were analyzed. The study confirmed that the classification system based on Raman spectroscopic features could effectively distinguish between remission and non-remission AML, achieving a prediction accuracy of 94.8%. Furthermore, significant differences were observed in metabolites including nucleic acids and lipids between the two patient groups, establishing a novel basis for developing treatment decisions utilizing Raman spectral fingerprint metabolism.

## Materials and methods

### Raman spectroscopy system

**FIGURE 1:**
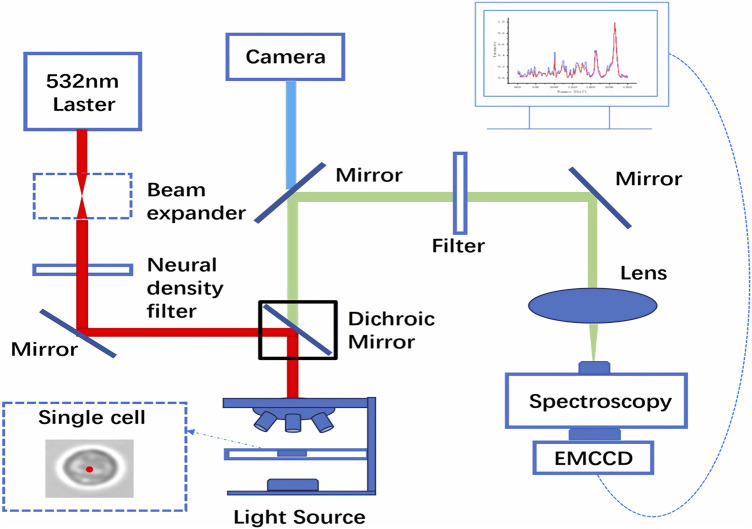
Structure diagram of Raman spectral system.

The instrumentation employed was a self-developed spontaneous Raman spectroscopy system (532 nm excitation source) (Figure 1), with detailed core optical specifications provided in Supplementary Table S1. The expanded collimated laser beam is focused onto the sample, and backscattered Raman signal is collected via one of two calibrated Olympus MPLANFLN objectives (10X/0.30 NA and 50X/0.80 NA for different measurement requirements). A 532 nm deep-cutoff rapid edge Raman Rayleigh rejection filter (Edmund Optics) was used to suppress Rayleigh background scattering prior to the signal entering the spectrometer’s 30–100 μm wide entrance slit. The signal was dispersed by a plane diffraction grating (1800 grooves/mm, 76 mm clear aperture, blazed at 532 nm for maximum diffraction efficiency) and detected by an electron-multiplying CCD (16 μm pixel size) cooled to −40 °C to minimize thermal noise and improve signal-to-noise ratio, enabling full-range Raman shift acquisition of 100–4000 cm^−1^. Spectral resolution characterization was performed via mercury lamp calibration to ensure scientific rigor, with an overall spectral resolution of <2 cm^−1^ achieved. Specifically, the adjacent mercury emission lines at 579.07 nm and 579.96 nm were mapped to CCD pixels 718 and 646, respectively, yielding a calculated pixel resolution of ∼0.028 nm. Wavenumber calibration was performed via linear interpolation of mercury line reference data, and calibration accuracy was validated before each measurement using the standard 520.7 cm^−1^ peak of single-crystal silicon. Three consecutive scans under identical conditions confirmed the deviation between measured and theoretical wavenumber was <2 cm^−1^, meeting performance requirements for clinical Raman analysis.

### Patients selection

In this study, patients diagnosed with AML and treated at Department of Hematology, the First Hospital of Jilin University, between April 2023 and June 2024 were enrolled. The diagnosis of the disease strictly adhered to the World Health Organization (WHO) 5th edition criteria for the classification of hematopoietic and lymphoid tissue tumors (Arber et al., 2022). All samples (BM) aspirates were obtained within a strictly defined at baseline (pre-treatment) before induction therapy. Raman spectroscopy analyses were completed within 24 h of sample collection to ensure preservation of cellular metabolic states. This early-phase sampling strategy targets the initial response dynamics of leukemia cells, enabling detection of primary resistance mechanisms emerging before clinical therapy. The patients were confirmed using a multi-parameter integrated diagnostic system, including (DiNardo et al., 2023): bone marrow (BM) cell morphology (Yeung and Radich, 2017); flow cytometry immunophenotypic analysis (Santoro et al., 2024); cytogenetic karyotype analysis (Döhner et al., 2022); molecular biological detection. Based on the inclusion criteria (age≥18 years, newly diagnosed AML, and no prior therapy) and exclusion criteria (presence of other malignancies, severe organ dysfunction, or secondary AML, a total of 18 eligible BM samples were included. The study included 18 patients with previously untreated *de novo* non-APL AML. Genetic subtypes were classified according to the 2022 WHO classification (Arber et al., 2022). All patients received cytarabine (ara-C)-based frontline induction therapy as we previous reported (Li et al., 2025), and treatment response was assessed per the 2022 ELN guidelines (Döhner et al., 2022) based on the proportion of BM blasts and peripheral blood recovery after induction chemotherapy. The patients were categorized into the CR (n = 9) and non-remission (NR, n = 9) groups, as shown in Table 1. This study was approved by the Medical Ethics Review Committee of the First Hospital of Jilin University (approval number: 2021-347), and all participants provided written informed consent prior to enrollment. The design and implementation of the study strictly adhered to the ethical guidelines set forth in the Declaration of Helsinki.

**TABLE 1:** Characteristics between CR and NR patients with AML.

Characteristics	CR	NR	*P* values
Age, median (range), y	45 (22–68)	50 (24–67)	0.846
Gender, Male (n, %)	5 (55.56%)	8 (88.89%)	0.014
CBC pre-treatment			
WBC (×10^9^/L)	10.46 ± 10.96	23.25 ± 26.58	0.218
HGB (g/L)	63.18 ± 26.75	71.33 ± 13.41	0.430
Platelets (×10^9^/L)	47.36 ± 37.61	66.00 ± 62.54	0.450
Blasts in BM (%)	67.61 ± 27.03	57.67 ± 21.63	0.341
Classification with WHO, n			
AML with mutated *TP53*	2	1	​
AML with *NPM1* mutation	2	3	​
AML with in-frame *CEBPA* ^bZIP^ mutations	1	1	​
AML with t (8; 21)(q22; q22.1)/*RUNX1*::*RUNX1T1*	1	1	​
AML with r-KMT2A	1	1	​
AML with MR gene mutations	0	1	​
AML with −7/del (7q)	1	1	​
AML, not otherwise specified	1	0	​

CR: complete remission; NR: non-remission; CBC:complete blood counts; WBC: white blood cells; HGB: hemoglobin; PB: peripheral blood; BM: bone marrow; WHO: world health organization.

### Specimen preparation and spectroscopic measurements

Initially, BM samples from newly diagnosed AML patients were processed using density gradient centrifugation (400×g for 30 min at room temperature) with a lymphocyte separation medium (Ficoll-Paque™ PLUS, density 1.077 g/mL) to isolate the mononuclear cell fraction at the interface. The resulting cell suspension was washed twice with PBS buffer and then adjusted to a concentration of 1 × 10^6^ cells/mL using a hemocytometer. Cell viability, confirmed by Trypan blue exclusion, was greater than 95%. Subsequently, cell suspensions were spotted onto standard glass slides (e.g., pathological-grade microscope slides glass). To maintain cell viability and physiological morphology, spectral acquisition was performed with the cells immersed in 1× phosphate-buffered saline (PBS) buffer, ensuring they remained in a hydrated state throughout the measurement. Cells were then screened using a micromanipulation platform. Specifically, under the guidance of bright-field imaging, qualified cells meeting pre-specified morphological criteria (15–20 μm in diameter, intact cell membrane) were randomly selected from each patient sample. A 2 μm diameter laser spot was positioned at the center of each qualified cell, and single point Raman spectra were acquired from the cell center with a laser power of 40 mW. To optimize signal to noise ratio (SNR) while minimizing laser exposure time, each cell’s spectrum was acquired by averaging the signal over 3 accumulations (scans) within a single measurement sequence, with an integration time of 15 s per scan. After spectral collection, bright-field imaging was re-assessed to confirm that the targeted cell positioning still conformed to our selection criteria.

### Spectral data processing and analysis

#### Initial processing

The initial spectra were subjected to four sequential preprocessing steps, with representative changes shown in Supplementary Figure S1. First, spectral denoising was performed using optimized Savitzky-Golay (S-G) filtering. After evaluating denoising performance by RMSE and comparing multiple parameters, third-order S-G filtering with a window size of 25 was chosen for its optimal noise suppression and spectral feature preservation. Second, the glass substrate background was removed using a discrete wavelet transform (DWT) based scale separation method. A reference glass spectrum was established from blank measurements (Supplementary Figure S2), and the substrate contribution was fitted and subtracted from cell spectra after DWT decomposition and reconstruction. Third, fluorescence baseline drift under 532 nm excitation was corrected using equal interval cubic spline interpolation combined with zero-order S-G filtering, with the sampling interval adjusted until fitting convergence. Finally, Min-Max normalization was applied to standardize spectral intensities into the range [0, 1], unifying signal scales and improving analytical stability. All spectral analyses focused on the 600–1800 cm^−1^ fingerprint region.

#### Multi-statistical analysis and classification of spectra

Multivariate statistical analysis was performed to construct the spectral feature analysis system. First, Principal Component Analysis (PCA) (Massei et al., 2024) was applied to reduce the dimensionality of the high-dimensional spectral dataset. The core principle of PCA is to project the original variables into an orthogonal principal component space along the direction of maximum variance, achieved through eigenvalue decomposition of the covariance matrix. In this study, PCA was combined with score matrix visualization to highlight the clustering trends of the samples. Subsequently, Linear Discriminant Analysis (LDA) was used to construct a discriminant function for the dimensionally reduced data. This algorithm defines discriminant boundaries with maximum inter-class separation in the compressed feature space, determining the optimal projection hyperplane. The PCA-LDA combination eliminates data redundancy through orthogonal dimensionality reduction, followed by supervised discriminant analysis, which enhances the robustness and interpretability of the classification model. This model was used to predict the CR and NR groups. Model performance was evaluated using Receiver Operating Characteristic (ROC) analysis (Park et al., 2004; Mandrekar, , 2010), which generates parametric curves of true and false positive rates by systematically varying the classification thresholds. This allowed for a quantitative assessment of the model’s predictive ability for the initial treatment response in AML patients. The AUC was calculated as the primary evaluation metric (Zam et al., 2021). To further enhance spectral resolution accuracy, Multivariate Curve Resolution-Alternating Least Squares (MCR-ALS) analysis in this study was performed using the MCR-ALS GUI 2.0 toolbox (Felten et al., 2015), which operates within the MATLAB environment,and was implemented following Felten et al.'s protocol (Felten et al., 2015) to construct spectral decomposition model. The number of components was determined by singular value decomposition (SVD) combined with PCA, where the elbow point of the singular value spectrum was used to separate chemical signals from noise. Initial estimates of pure component spectra were obtained using the SIMPLISMA algorithm with a noise level of 10%. The alternating least squares optimization was conducted with a maximum of 50 iterations and a convergence criterion of 0.1% relative change in the residual standard deviation. Non-negativity constraints were applied to both spectral and concentration profiles. The final model was selected based on quantitative fit statistics and the chemical interpretability of the resolved components.

### Statistical analysis

All statistical analyses were performed using MATLAB 2019b (MathWorks, United States), while visualization and analysis of the feature spectra were carried out using Origin 2018 (OriginLab, United States). The data were expressed as mean ± standard deviation. For pairwise comparisons, an unpaired Student’s t-test was applied when the data exhibited a normal distribution and homogeneity of variance; otherwise, the Mann-Whitney U test was utilized. Statistical significance was defined as a *P* value < 0.05 across all analyses. *P* values were denoted as follows: *P < 0.05; **P < 0.01; ***P < 0.001; ****P < 0.0001.

## Results

### Association of Raman spectral profiles with the initial treatment response of AML

**FIGURE 2:**
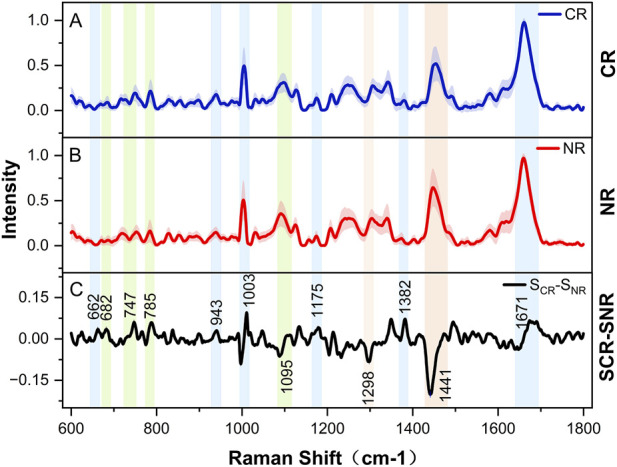
Mean Raman spectra of AML cells from CR and NR patients. **(A)** Mean Raman spectrum (blue solid line)and associated standard deviation (shaded band) for the complete response group (CR; n = 335). **(B)** Mean Raman spectrum (red solid line) and associated standard deviation (shaded band) for the non-remission group (NR; n = 322). **(C)** Differential spectrum derived from the mean Raman spectra of CR and NR groups. Statistically significant peaks in the differential spectrum are highlighted with background colors corresponding to primary biomolecular assignments: green (nucleic acids), blue (amino acids), and orange (lipids).

Firstly, this study investigated the association between Raman spectral profiles and the response to initial induction therapy in patients with *de novo* AML. The cohort consisted of nine patients who achieved CR and NR controls. Table 1 presented the clinical characteristics of these patients. As expected, no significant differences were observed between the two groups in terms of demographic features and other clinical parameters (all *P* > 0.05). This balanced distribution effectively mitigated potential confounding factors, thereby minimizing their influence on the spectral characteristics. The analysis utilized a high signal-to-noise ratio Raman spectral dataset, encompassing single-cell spectra from bone marrow (BM) leukemia samples in the CR group (n = 335) and NR group (n = 322). The average spectra for both groups, generated using a spectral averaging algorithm, exhibited substantial overlap within the 600–1800 cm^−1^ fingerprint region. Notably, considerable intra-group spectral heterogeneity was observed in both the CR and NR groups, and the mean values and standard deviations of all characteristic peaks were calculated (as indicated by the shaded areas in Figures 2A,B). Differential spectroscopic analysis identified significant peaks that distinguish the two groups, which correspond primarily to the characteristic vibrational modes of nucleic acids, amino acids, and lipids (Table 2) (Gaba et al., 2022; Cutshaw et al., 2023; Bai et al., 2020; Liu et al., 2017; Talari et al., 2014). These differentially expressed peaks remained consistently detectable despite the high intra-group spectral variability. The inter-group intensity variations remained relatively minor (Figure 2C), suggesting a high degree of biological homogeneity in the metabolomic profiles of AML patients at baseline.

**TABLE 2:** Major Raman spectral peaks and corresponding components in AML cells.

The peak position/cm^-1^	Biomolecules and vibrationalassignments	Molecules
662	Glutathione	Proteins
682	Ring breathing modes in the DNA bases G (ring breathing modes in the DNA bases)	Nucleic acids
747	T (ring breathing mode of DNA/RNA)	Nucleic acids
785	U, T, C (ring breathing modes in the DNA/RNA bases) Backbone O-P-O	Nucleic acids
943	Skeletal modes (polysaccharides, amylose)	Carbohydrate
1003	Phenylalanine, C C skeletal	Proteins
1095	Lipid Phosphodioxy group (PO2- in nucleic acids)	Nucleic acids
1175	Cytosine, Guanine	Nucleic acids
1235	Amide III	Proteins
1298	Palmitic acid Acyl chains Fatty acids	Lipids
1382	CH3 band	Lipids
1441	C-H vibration (proteins)	Proteins/Lipids
1671	Amide I vibration mode of structural proteins/cholesteol	Proteins/Lipid

**FIGURE 3:**
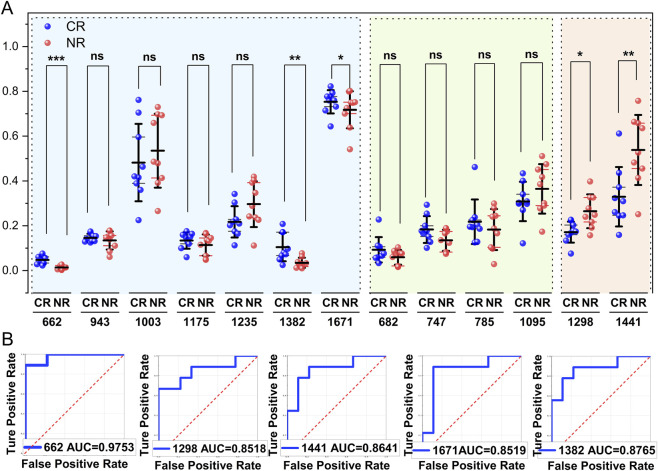
Scatter plot of the peak intensity distribution of the average spectra of the patients. **(A)** Different peak intensity distribution between the CR and NR groups. The green backdrop represents nucleic acid, blue represents protein, and orange represents lipid. *P < 0.05,*P < 0.01,***P < 0.001 (unpaired t-test or mann-whitney test). **(B)** Different ROC curve between the CR and NR groups.

Base on the Raman vibrational mode attribution system, the characteristic upregulated spectral bands in the CR group encompassed: 682 cm^−1^ (antisymmetric stretching vibration of DNA phosphodiesters), 747 cm^−1^ (thymine ring breathing vibration), 785 cm^−1^ (symmetric stretching vibration of phosphodiesters), and other nucleic acid-related peaks. Additionally, notable amino acid metabolism markers were detected at 662 cm^−1^ (glutathione S-S stretching vibration), 1003 cm^−1^ (phenylalanine ring breathing vibration), and 1175 cm^−1^ (tyrosine C-H plane bending vibration). Intensity disparities at 943 cm^−1^ (glucose β-1,4-glucosidic bond vibration) and 1745 cm^−1^ (acyl C=O stretching vibration), both related to energy metabolism, suggested heterogeneity in glycolysis and tricarboxylic acid cycle pathway activities. Remarkably, the drug-resistant group exhibited significantly higher levels at 1298 cm^-1^ (cholesterol C-C skeleton twisting vibration) and 1441 cm^−1^ (lipid chain CH_2_ scissoring vibration), implying that the drug-resistant phenotype may be linked to membrane lipid raft remodeling and cholesterol homeostasis imbalance (Floren and Gillette, 2021). To address potential pseudoreplication and inflated statistical significance arising from treating multiple spectra within the same patient as independent observations, patient-level aggregates data were reanalyzed. Using these patient-level spectra, scatter plots depicting the intensity distribution of characteristic peaks from the differential Raman spectra were generated, as well as the ROC curve analysis and AUC values, followed by inter-group comparative analysis (Figures 3A,B). The results demonstrated that the intensity differences between the two groups were significant (P < 0.05) at characteristic peak positions of 662 cm^−1^, 1298 cm^−1^, 1382 cm^−1^, 1441 cm^−1^, and 1671 cm^−1^. While other spectral features exhibited trends suggestive of group differences, these did not reach statistical significance due to the limitation potentially attributable to sample size constraints. ROC curve analysis demonstrated outstanding diagnostic performance across all characteristic peaks, with all AUC values exceeding 0.85. Particularly, the peak at 662 cm^−1^ achieved superiority discrimination (AUC = 0.9753). The 662 cm^−1^ peak corresponds to the vibrational model of reduced glutathione, a key regulator of oxidative stress in leukemia, thereby highlighting its prodicative significance (Ma et al., 2023). Conversely, the spectral overlap of multiple molecular constituents, including proteins and lipids, at the 1441 cm^−1^ peak (Wang et al., 2023) underscores the intrinsic limitations of traditional univariate spectral analysis for biomolecular identification. These findings uncover the link between Raman spectral signatures and the initial treatment response of AML. Key peaks for nucleic acids, amino acids, and lipids differ significantly between CR and NR groups, but deep learning is needed to overcome traditional spectral analysis limitations.

### PCA-LDA cluster analysis of Raman spectrum

**FIGURE 4:**
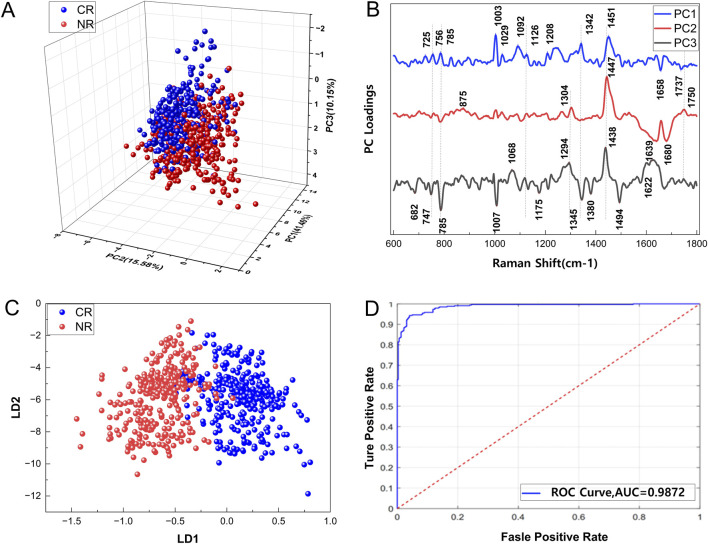
Raman spectral PCA-LDA cluster analysis of different reaction types in newly diagnosed AML patients. **(A)** The first three PC scores in PCA spatial scatter cluster analysis diagram. **(B)** The PC loads for the first three principal components. **(C)** Scatter cluster analysis diagram of LDA. **(D)** ROC curve of the PCA-LDA model.

To identify key spectral differences between groups with all patients, PCA was performed on spectral data from CR and NR AML patients. PCA effectively segregated CR and NR AML cells by reducing 1761 dimensional spectral data into independent principal components (PCs). Distribution and loading plots of the top three PCs are shown (Figures 4A,B). PC1 accounted for 41.46% of total spectral variance, with its loading spectrum revealing key peak distributions. Prominent positive loadings occurred at: 725 cm^−1^ (nucleic acids), 756 cm^−1^ (tryptophan), 785 cm^−1^ (nucleic acids), 1003/1029 cm^−1^ (phenylalanine), 1092 cm^−1^ (proteins), 1126/1208 cm^−1^ (lipids/proteins), 1342 cm^−1^ (nucleic acids/proteins), 1658 cm^−1^ (amide I), and 1737 cm^−1^ (lipid ester). PC2 exhibited positive loadings at 875 cm^−1^ (proteins), 1304 cm^−1^ (nucleic acids/lipids), 1447 cm^−1^ (lipids), and 1750 cm^−1^ (lipids). PC3 demonstrated positive loadings at 1068 cm^−1^ (lipids), 1294 cm^−1^ (proteins), and 1441 cm^−1^ (lipids), with significant negative loadings at 682/747/1007/1175/1342/1494 cm^−1^ (nucleic acids), 1007 cm^−1^ (proteins), 1380 cm^−1^ (glucosamine), and 1750 cm^−1^ (lipids) (peak attributions detailed in Supplementary Table S2). The first three PCs collectively explained 67.20% of cumulative variance, yielding 75.95% test-set classification accuracy. To optimize performance, PCs were selected based on >90% cumulative variance contribution. Incorporating the top 24 PCs (90.1% variance) as feature inputs for the supervised classification model improved accuracy to 93.5% (Supplementary Figure S3), with additional components providing no significant enhancement. LDA was employed for model development. To validate the classification performance of the model and prevent potential overfitting, ten-fold cross-validation was implemented to optimize sensitivity and specificity metrics. Model performance was quantitatively evaluated using ROC curves and AUC. LDA score plots (LD1 vs. LD2) showed clear separation between CR and NR groups, illustrating the model’s high discriminative performance (Figure 4C). To identify the key spectral features driving group separation, we analyzed the absolute normalized weights of the LDA eigenvectors, only LD1captures meaningful between-group variance. The cumulative explained variance confirms that LD1 alone accounts for 100% of the total between-group variance, establishing it as the statistically meaningful discriminative dimension. As shown in the Supplementary Figure S4 illustrating PC contributions to LD1, the top three principal components (PCs) with the highest contributions are PC5 (8.79%), PC14 (8.15%), and PC13 (7.35%), which together account for 24.3% of the total normalized weight. This distributed contribution pattern indicates that the discriminatory information is not concentrated in a single PC, but instead distributed across multiple components, reflecting the complex and multivariate nature of the spectral differences between the two groups. Of 335 CR bone marrow (BM) spectra, 35 were misclassified as NR. For 322 NR spectra, the model achieved 89.55% sensitivity and 90.31% specificity, with 89.34% overall cross-validated accuracy. The ROC curve demonstrated excellent classification (AUC = 0.9627; Figure 4D).

In the subsequent analysis, to further present the patient-level analytical results, we performed PCA-LDA analysis on five patients with confirmed NPM1 mutations (two in remission and three in non-remission), to investigate whether the presence of shared mutations among AML patients influences analytical outcomes. As illustrated in Supplementary Figure S5A, the three-dimensional PCA scatter plot based on the first three principal components (PC1, PC2, and PC3) revealed distinct clustering patterns associated with therapeutic response. Patients who achieved CR were predominantly clustered together, represented in blue and green, whereas non-remission NR patients, depicted in red, orange, and pink, formed a separate and distinct cluster. This separation suggests that even among individuals sharing the same NPM1 mutational background, patients with distinct clinical outcomes can be distinguished based on their molecular features. Furthermore, the three-dimensional LDA score plot in Supplementary Figure S5B demonstrated clear separation between patient groups, with individuals exhibiting similar treatment responses tending to cluster together. These findings indicate that LDA effectively captures variation associated with treatment response and supports the potential of such multivariate approaches for prognostic stratification within genetically homogeneous subgroups of acute myeloid leukemia. Moreover, Raman spectroscopy may be capable of capturing spectral features associated with shared resistance mechanisms, even in the context of similar genetic backgrounds.

### Verification of the predictive values with external validation in PCA-LDA model

**FIGURE 5:**
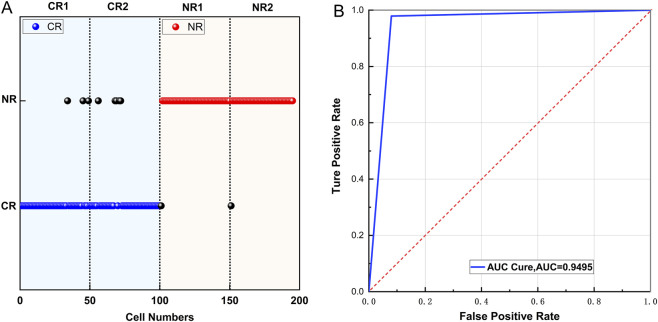
Validation of treatment response prediction model for newly diagnosed AML patients based on linear discriminant analysis (LDA). **(A)** Blinded validation of four newly enrolled patients with newly diagnosed AML (CR = 2, NR = 2); each independent sample contains 45 to 50 high-quality spectra, with blue scattered for CR, red scattered for NR, and black scattered for misdetermined spectra. **(B)** ROC curve of validation of treatment response prediction model.

To assess the statistical validity of the model, a Permutation Test was employed. The sample labels were randomly permuted 1000 times, and the LDA model was reconstructed with each permutation. The AUC was calculated for each iteration, generating a null distribution (Supplementary Figure S6). These findings statistically confirmed the true correlation between spectral features and treatment response, effectively ruling out false positives arising from random variation. For assessing the diagnostic and predicitve performance of the model, four independent validation samples, none of which were included in the model training process, were utilized (Figure 5A). This validation set comprised two patients with CR and two with NR. A total of 45–55 Raman spectra per patient (195 spectra in total) were obtained from AML cells. The integrated PCA-LDA classification model achieved 100% accuracy at the population level across all four patients (4/4). The individual spectral classification results were as follows: for CR patients, Patient 1 achieved an accuracy of 92.9% (52/56), and Patient 2 achieved 92.1% (41/45), resulting in an overall CR spectrum recognition accuracy of 92.0% (92/100); for NR patients, Patient 1 had an accuracy of 98.0% (49/50), and Patient 2 had 97.8% (44/45), yielding an overall NR identification accuracy of 97.9% (93/95). The overall classification accuracy of this model was 94.8% (185/195), with a sensitivity of 97.9%, a specificity of 92.0%, and an AUC of 0.9495 (Table 3; Figure 5B). These results demonstrate that dimensionality reduced spectral data represents a promising predictive tool for initial AML treatment response.

**TABLE 3:** Confusion matrix obtained for PCA-LDA model.

Prediction	Actual		Accuracy	Sensitivity	Specificity
	NR	CR			
CR	6	94	94.80%	97.90%	92.00%
NR	93	2	​	​	​

PCA: principal component analysis; LDA: linear discriminant analysis; CR: complete remission; NR: non-remission.

### MCR-ALS analyses of various compositions linked to therapeutic response

**FIGURE 6:**
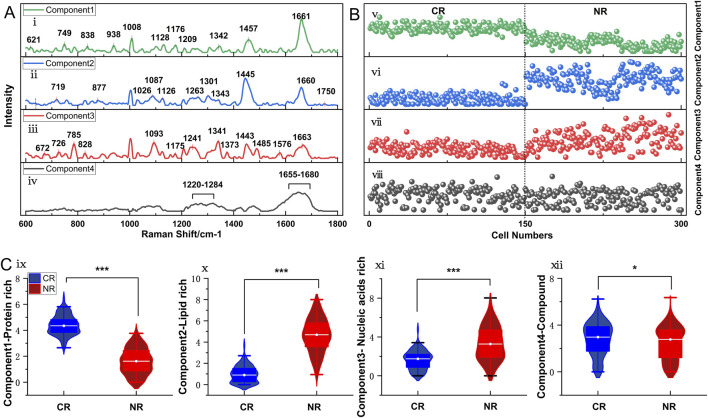
Analysis of Raman spectra from AML cells of newly diagnosed patients with different treatment responses using MCR-ALS. **(A)** Decomposition into four spectral components. **(B)** Concentration values corresponding to the four components. **(C)** Histograms of the average abundance of the three components. P-values obtained from t-tests to determine if there are significant differences between the two groups. *P < 0.05, ***P < 0.001 (unpaired t-test or mann-whitney test).

For dissecting the concentration and substances underlying differential Raman spectra, MCR and ALS analyses were employed. The established MCR model explained 96.90% of spectral variance, successfully resolving four distinct pure components with specific biophysical implications (Figure 6A). These components exhibited characteristic spectral profiles corresponding to: Component 1 exhibited typical protein-specific bands: 621 cm^−1^, 749 cm^−1^, 938 cm^−1^, 1008 cm^−1^, 1176 cm^−1^ and 1342 cm^−1^, 1457 cm^−1^, 838 cm^−1^, 1128 cm^−1^, and 1661 cm^−1^. Component 2 contained spectral features indicative of lipids 719 cm^−1^, 877 cm^−1^, 1087 cm^−1^, 1301 cm^−1^, 1445 cm^−1^, 1660 cm^−1^, and and carbohydrates with 1026 cm^−1^ and 1126 cm^−1^ (belonging to C-O-H vibration modes of glycogen and disaccharides). Component 3 displayed nucleic acid-specific signals: 627 cm^−1^, 726 cm^−1^, 785 cm^−1^, 828 cm^−1^, 1093 cm^−1^ (phosphate group symmetric stretching vibration), and characteristic peaks at 1485 cm^−1^ and 1576 cm^−1^. Component 4 represented a mixed vibration peak, reflecting contributions from nucleic acids, amino acids, and lipids. The attribution of these spectral peak bands is shown in Supplementary Table S3. Notably, Raman bands (like 749 cm^−1^ and 938 cm^−1^) can originate from multiple biomolecules. Such dual interpretation aligns with spectral degeneracy in biological systems and the complementary roles of multivariate resolution and difference spectroscopy.

To assess the biomolecular composition differences between groups, the MCR-ALS scores of the CR and NR groups were systematically compared using scatter plots and subjected to statistical analysis (Figure 6B,C). Analysis revealed distinct molecular signatures: The CR group exhibited a significantly increased relative abundance of the protein component (Component 1; *P* < 0.05), with its spectral intensity positively correlating with therapeutic responsiveness. Conversely, the NR group demonstrated a marked elevation in the lipid-carbohydrate component (Component 2; *P* < 0.05). Content analysis based on nucleic acid spectral peaks (Component 3) indicated significantly lower nucleic acid levels in CR samples compared to NR samples (*P* < 0.05), potentially reflecting differential cell proliferation rates. The mixed vibrational component (Component 4) showed a marginally significant difference between groups (*P* = 0.037), suggesting its potential role in reflecting core metabolic processes associated with cellular homeostasis.

Three-dimensional density maps visualize the spatial distribution of standardized MCR-ALS scores for AML cells with the first three component (protein, lipid, and nucleic acid abundance) in CR versus NR groups (Supplementary Figure S7). The component loadings define a 3D coordinate system wherein their relative spatial orientations reveal distinct distribution patterns of MCR-ALS scores. This spatial differentiation within the pure-component MCR-ALS loading subspace provides a basis for exploring the potential to discriminate therapeutic responses through multivariate component analysis.

## Discussion

This study suggests that RS was applied to predict the efficacy of the induction chemotherapy AML. By performing PCA-LDA classification modeling and MCR-ALS pure component resolution, spectral fingerprints differences between the CR group and NR group, and the predictive performance of the model was validated in an independent test set.

RS offers unique value for AML therapeutic response prediction. Current AML response prediction mainly relies on molecular genetic stratification systems, including karyotyping, mutation detection of genes such as *NPM1, CEBPA* and *TP53,* and ELN risk stratification (Döhner et al., 2022; Arber et al., 2022). However, these methods are limited by long turnaround times and high costs, and patients with identical genetic backgrounds may still exhibit drastically different treatment responses. As a label-free, non-invasive technique that rapidly acquires molecular fingerprint information at the single-cell level (Cheng et al., 2022; Leszczenko et al., 2024), Raman spectroscopy has shown promising potential in leukemia diagnosis and classification (Vanna et al., 2024; Managò et al., 2016; González-Solís et al., 2014). The novelty of this study lies in extending Raman spectroscopic analysis from disease diagnosis to the field of clinical treatment response prediction. This multiparameter, label-free detection approach overcomes the limitations inherent in single-marker assays and enables a comprehensive molecular fingerprint information underlying chemotherapy resistance in acute myeloid leukemia. Importantly, such heterogeneity remains undetectable using conventional methods within a single, unmodified cell system.

The key findings of this study are as follows. Firstly, differences in redox metabolism represent the strongest marker distinguishing the two groups. The Raman peak at 662 cm^−1^, associated with glutathione, showed a significant difference at the patient level (*P* < 0.05), with an AUC of up to 0.9753. In drug resistant AML cells, the metabolism of glutathione is abnormal. Through the ABCC1 transporter, glutathione and its conjugates are excreted, thereby alleviating the oxidative stress induced by chemotherapy drugs (Ebner et al., 2023). Leukemia stem cells can reduce oxidative stress via MRP1-mediated GSH efflux (Niu et al., 2022). Second, in this study, variations in Raman spectral intensities at 1298 cm^−1^ and 1441 cm^−1^ indicated alterations in lipid composition (*P* < 0.05). Lipid metabolism supports membrane biosynthesis and energy requirements in leukemia cells, and inhibiting lipid synthesis or oxidation can induce apoptosis and improve chemotherapy sensitivity. MCR-ALS analysis further confirmed an enrichment of lipid components in drug-resistant patients (*P* < 0.05). These results strongly suggest a close link between the drug-resistant phenotype and lipid metabolic reprogramming, which is highly consistent with findings reported by Managò et al. in B-cell acute lymphoblastic leukemia (B-ALL) (Managò et al., 2016). Lipid remodeling is another mark of chemotherapy-resistant AML (Farge et al., 2023). Targeting lipid metabolism has shown therapeutic promise. Inhibition of fatty acid synthase (FASN) (Humbert et al., 2021) or carnitine palmitoyltransferase 1A (CPT1A) (Shi et al., 2016) leads to synthetic lethality in lipid-dependent AML clones (Pandyra et al., 2014). Clinical trials have reported a 75% overall response rate in relapsed/refractory AML patients treated with statins in combination with salvage chemotherapy (Advani et al., 2014). At the same time, MCR-ALS analysis showed that the nucleic acid component (Component 3) abundance in the NR group was significantly higher (*P* < 0.05). The difference in nucleic acid content may reflect heterogeneity in proliferation activity and cell cycle distribution between the leukemia cells of the two groups (Mojidra et al., 2021; Yabushita and Goyama, 2025). MCR-ALS enabled the extraction of four chemically distinct and biologically interpretable spectral component, thereby providing direct spectroscopic evidence of multidimensional metabolic reprogramming in NR cells. Furthermore, the integrated PCA-LDA model coupled with RS demonstrated robust discriminative power (AUC = 96.27%) by decoding overlapping vibrational signatures within the fingerprint region. External validation achieved a 94.8% accuracy in CR prediction, positioning RS as a rapid, label-free alternative to resource-intensive test.

However, this study enrolled 18 AML patients for model development and 4 for external validation, critically, the cohort did not exclude individuals with pre-existing metabolic disorders (e.g., diabetes, hyperlipidemia) or control for other confounding factors, potentially biasing outcomes. As the primary constraint is the small sample size, which limits statistical power and generalizability. Future studies must expand multi-center cohorts with standardized protocols to enhance clinical scalability and mitigate batch effects. Molecular assignment of Raman peaks relies on established vibrational mode databases, and the multi-molecule overlapping nature of characteristic peaks introduces uncertainty in precise molecular identification. Further cross-validation using mass spectrometry, metabolomics or other omics techniques is therefore warranted, and the elucidation of the specific underlying pathways will require further genomic or biochemical validation.

In conclusion, this study provides proof of concept for the use of RS in predicting AML treatment response. As a rapid, non-destructive optical detection technique, RS offers broad prospects for clinical application. However, its integration into routine clinical practice requires further validation and methodological optimization. Future studies should explore the combination of RS with other diagnostic modalities, as well as its potential role in monitoring treatment response, to provide stronger support for the diagnosis, prognosis, and personalized management of AML.

## Data Availability

The original contributions presented in the study are included in the article/Supplementary Material, further inquiries can be directed to the corresponding authors.
