# Supplementary material for: Single cell Raman spectroscopic profiles predict treatment responses in patients with de novo acute myeloid leukemia
Source: Front Cell Dev Biol. 2026 Apr 28;14:1767226. doi: 10.3389/fcell.2026.1767226 (PMC13161155; doi:10.3389/fcell.2026.1767226)
Supplement: Supplementary file 1 [file DataSheet1.docx]

**Supplementary materials**

**Single cell raman spectroscopic profiles predict treatment responses in patients with de novo acute myeloid leukemia**

Ming Zhang ^1, 2^, Long Su ^1, 2^, Wei Han ^1, 2^, Fei Song ^1, 2^, Yu Fu ^1, 2^, Ming-Bo Chi ^3^, Xing Chen ^3^, Yi-Hui Wu ^3,^ *, Su-Jun Gao ^1, 2^^,^ *

^1^ Department of Hematology, The First Hospital of Jilin University; Changchun, 130021, China.

^2^ Key Laboratory of Hematology Precision Medicine of Jilin Province, The First Hospital of Jilin University; Changchun, 130021, China.

^3^ Changchun Institute of Optics, Fine Mechanics and Physics, Chinese Academy of Sciences, Changchun 130033, China

*Correspondence to:

Sujun Gao at sjgao@jlu.edu.cn. Department of Hematology, The First Hospital of Jilin University; Changchun 130021, China. Tel.: +86-431-88782842; fax: +86-431-88786134.

Yi-Hui Wu at yihuiwu@ciomp.ac.cn.Changchun Institute of Optics, Fine Mechanics and Physics, Chinese Academy of Sciences, Changchun 130033, China. Tel.: +86-431-85686367; fax: +86-431-85682346.

**Supplementary methods**

***Initial processing***

The initial spectral preprocessing mainly includes four successive steps: spectral denoising using optimized Savitzky-Golay filtering, glass substrate background removal via discrete wavelet transform, fluorescence baseline correction by cubic spline interpolation coupled with zero-order Savitzky-Golay filtering, and data standardization through Min-Max normalization. A representative comparison showing the evolution from raw spectra to preprocessed spectra is presented in Supplementary Fig.1. Firstly, denoising performance was evaluated using the root-mean-square error (RMSE), defined as

$$\text{RMSE}\text{=}\sqrt{\frac{\sum_{\text{n}} \text{(}\text{y}_{\text{c}}\text{-}\text{y}_{\text{a}}\text{)}^{\text{2}}}{\text{n}}}$$

where y_c_ and y_a_ represent the corrected and original spectral intensities, respectively. After comparing sliding window averaging and Savitzky-Golay (S-G)filtering with different parameters, S-G filtering with a third-order polynomial and a window size of 25 was adopted, as it yielded the minimum RMSE and effectively preserved spectral features while suppressing noise.

For glass substrate background removal, a discrete wavelet transform (DWT)-based scale separation method was employed. The continuous wavelet transform is given by

$$\text{WT}\text{(}\text{α}\text{,}\text{τ}\text{)=}\frac{\text{1}}{\sqrt{\text{a}}}\int_{\text{-∞}}^{\text{∞}} \text{f}\text{(}\text{t}\text{)*}\text{ψ}\left( \frac{\text{t}\text{-}\text{τ}}{\text{a}} \right)\text{dt}$$

Blank glass spectra were collected under the same experimental parameters as cell measurements, then denoised, normalized, and averaged to generate a glass reference spectrum(Supplementary Fig.2). Both cell spectra and the glass reference spectrum were decomposed to the same DWT levels, and approximate coefficients above a critical scale were set to zero for preliminary scale separation. After wavelet reconstruction, the contribution coefficient of the glass component was determined by linear fitting, and the substrate-free spectrum was obtained by subtracting the product of the glass reference spectrum and the contribution coefficient from the raw cell spectrum.

Fluorescence baseline drift under 532 nm excitation was corrected using equal-interval cubic spline interpolation combined with zero-order S-G filtering. Starting from an interval of 2, the sampling interval was gradually increased until the fitting converged, and local minima from adjacent fitting results were extracted to estimate the baseline. Zero-order S-G filtering with a window ranging from 2 to the final converged interval was then applied to separate the fluorescence background.

Finally, Min–Max normalization was used for data standardization according to

$$\text{X'}\text{=}\frac{\text{(}\text{X}\text{-}\text{X}_{\text{min}}\text{)}}{\text{(}\text{X}_{\text{max}}\text{-}\text{X}_{\text{min}}\text{)}}$$

where X represents the original Raman intensity, and X' is the characteristic value after data normalization, X_max_ and X_min_ are the maximum and minimum intensities of a single spectrum, and is the normalized value constrained to the range [0, 1]. This procedure unifies the intensity scale, reduces signal fluctuations from external disturbances, and enhances the stability of subsequent data analysis.The spectral features were focused on the 600-1800 cm⁻¹ fingerprint region for comparison and analysis. Data pre-processing was performed using MATLAB 2019b (MathWorks, USA), while visualization and analysis of the feature spectra were carried out using Origin 2018 (OriginLab, USA).

**Supplementary figures and tables**





**Fig. S1 Representative spectra showing the sequential preprocessing pipeline for Raman data.**

1. Raw spectrum; B. Spectrum after noise removal; C. Spectrum after glass substrate signal removal; D. Spectrum after fluorescence background removal and baseline correction; E. Final normalized Raman spectrum after complete preprocessing.


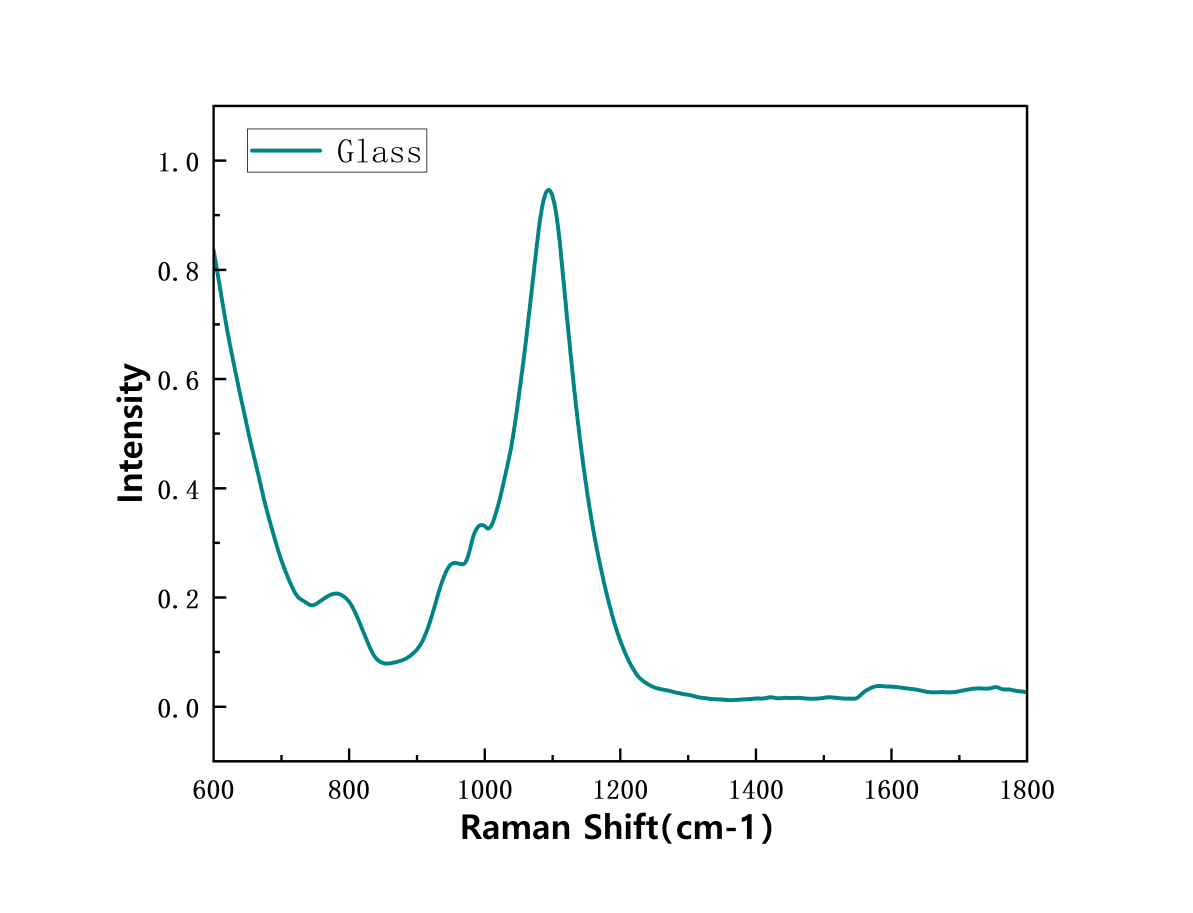


**Fig. S2 The spectral curve of glass.**

**
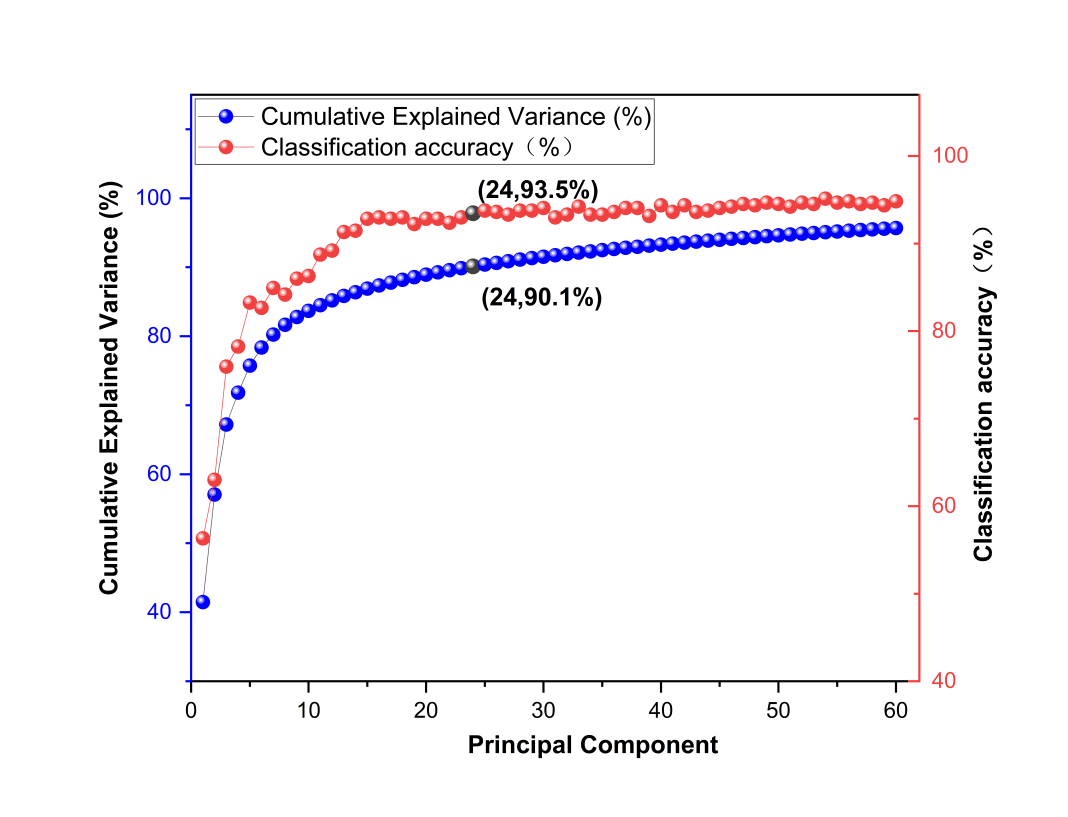
**

**Fig. S3** The blue curve is the cumulative explained variance of different PCs The red curve is classification accuracy of LDA and variance ratio under correspondence PC numbers.


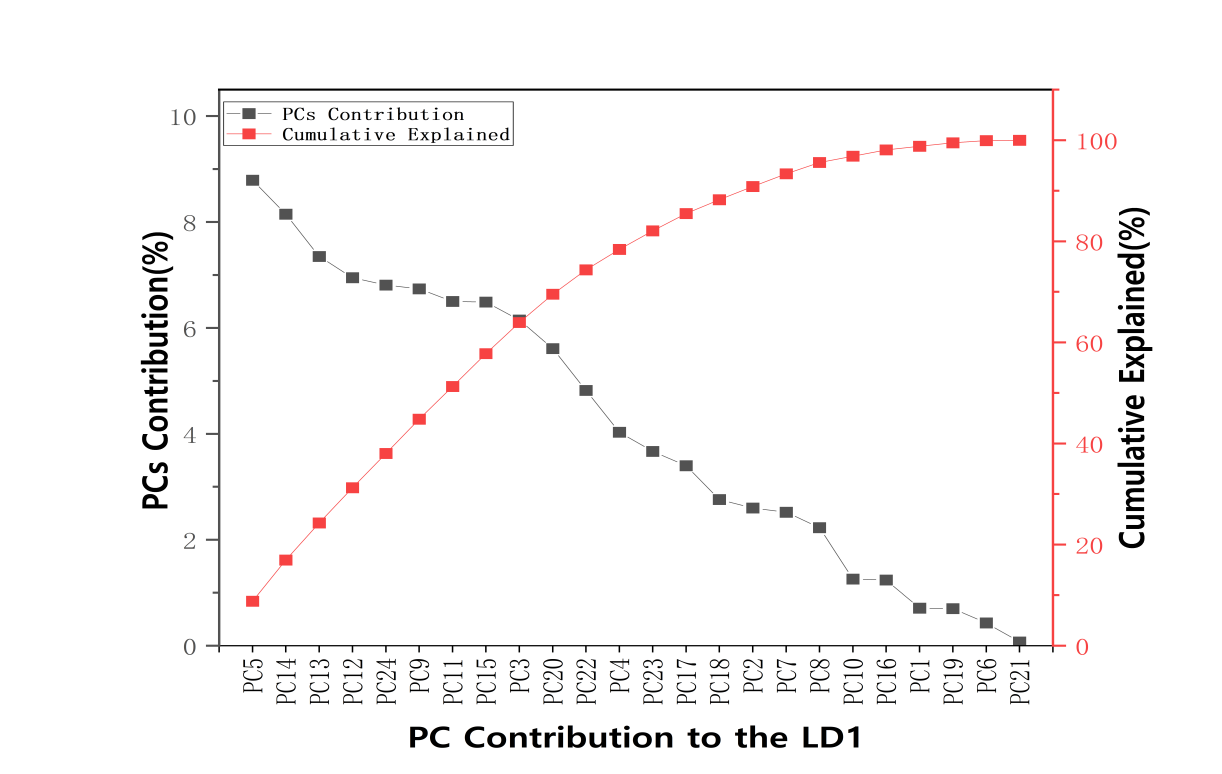


**Fig. S4 Principal component (PC) contributions to linear discriminant 1 (LD1).**

Black squares represent the percentage contribution of individual principal components (PCs) to linear discriminant 1 (LD1) (left y-axis), while red squares indicate the cumulative percentage of explained discriminant weight by ordered PCs (right y-axis).


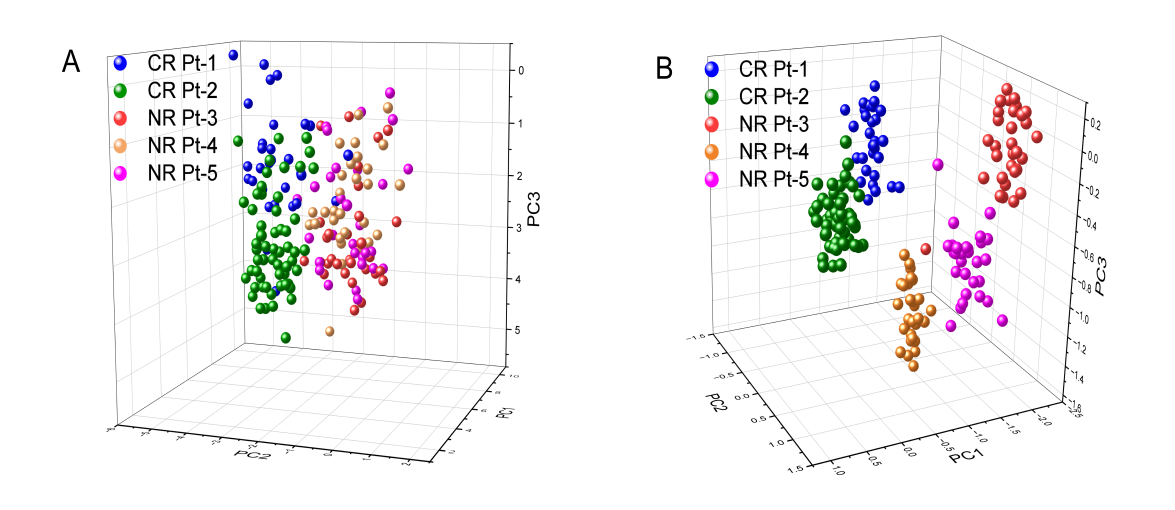


**Fig. S5** **PCA-LDA scatter plot of NPM1-mutated AML patients**

A.PCA scatter plot based on PC1 and PC2. CR (blue and green) and NR (red, orange, and pink) patients form separate clusters.B.LDA score plot (LD1 vs. LD2) showing clear separation between CR and NR groups, with patients clustering according to treatment outcome


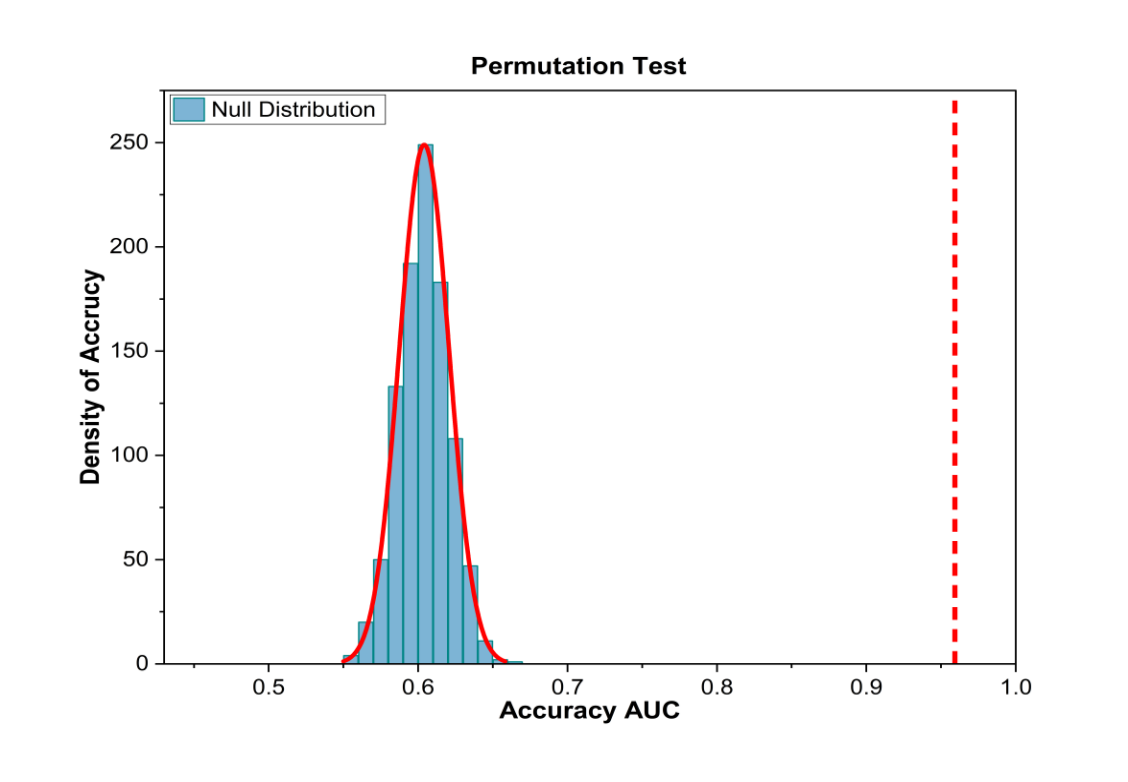


**Fig. S6** Applied to the AUC histogram in the permutation test analysis of the PCA- LDA mutation detection model, the red vertical line corresponds to the model AUC when correctly labeled.


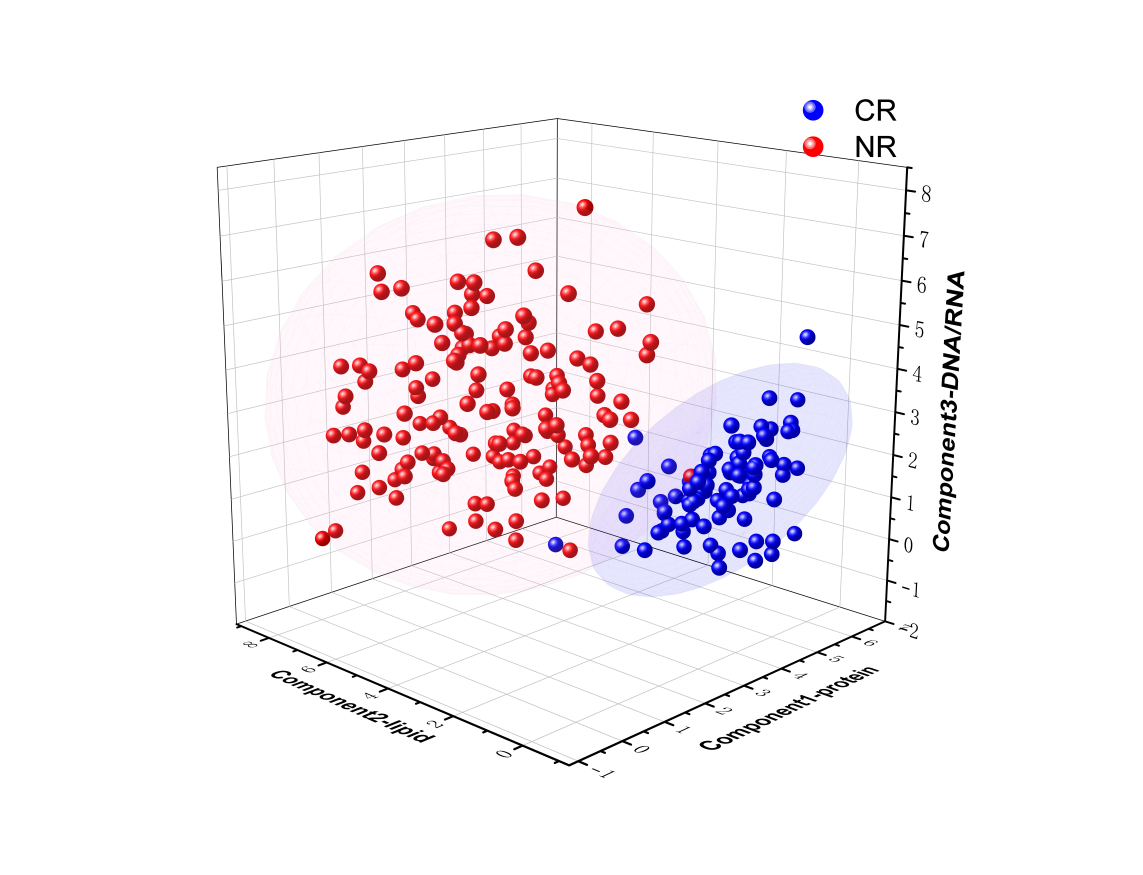


**Fig. S7** Three-dimensional scatter plots of MCR first three abundances: Normalized fractional distribution density plots of protein-rich, lipid-rich and nucleo-rich component loads for CR group (blue) and NR group (red).

Table S1. Specifications of optical components in the Raman spectrometer

| Optical Component | Parameter | Symbol | Value |
| --- | --- | --- | --- |
| Entrance slit | Width | a | 30−100 μm |
| Collimating mirror | Focal length | f_1_ | 560mm |
|  | Clear aperture | D_1_ | 80mm |
| Diffraction grating | Groove density | n | 1800 lines/mm |
|  | Width | W | 76 mm |
| Focusing mirror | Focal length | f_2_ | 388.5 mm |
|  | Clear aperture | D_2_ | 80mm |
| Plane reflection mirro | Clear aperture | D_3_ | 40mm |
| CCD detector | Pixel size | p | 16μm |

Table S2. Peak positions and assignments of major Raman spectral peaks observed in Leukemia cells in PCs

| The peak position/cm^-1^ | Biomolecules and vibrational assignments | Biological molecules |
| --- | --- | --- |
| 682 | G(ring breating mode of DNA) | Nucleic acids |
| 725 | A: ring breathing | Nucleic acids |
| 747 | T(ring breating mode of DNA/RNA) | Nucleic acids |
| 756 | Symmetric breathing tryptophan | Proteins |
| 785 | U,T,C(ring breating modes in DNA/RAN bases) Backbone O-P-O | Nucleic acids |
| 875 | Trptophan | Proteins |
| 1003 | Phenylalanine, C-C skeletal | Proteins |
| 1007 | Phenylalanine | Proteins |
| 1029 | Phenylalanine, C-C skeletal | Proteins |
| 1068 | Skeletal C-C strech | Lipids |
| 1092 | C-C sretching of protein,  O-P-O stretching | Proteins |
| 1126 | Protein, Phospholipids C-Ctr | Proteins/Lipids |
| 1175 | Cytosine, Guanine | Nucleic acids |
| 1208 | Trptophan, Phospholipid C-H | Proteins/Lipids |
| 1235 | Amide III | Proteins |
| 1294 | Methylene twisting | Proteins |
| 1298 | Palmitic acid, Acyl chains, Fatty acids | Lipids |
| 1304 | CH2 deformation, Adenine, Cytosine | Lipids/Nucleic acids |
| 1342 | A, G, C-H | Nucleic acids/Proteins |
| 1345 | Collagen, Tryptophan, Phospholipid | Proteins, Lipids |
| 1380 | Glucosamine | Carbohydrates |
| 1441 | C-H vibration(proteins/lipids) | Proteins/Lipids |
| 1447 | Phospholipid, C-H scissor in CH2 | Lipids |
| 1451 | CH2 bending of lipids, Proteins, Phospholipids (CH2/CH3), Amino acids | Proteins/Lipids |
| 1494 | Adenine, Guanine, C-H deformation | Nucleic acids |
| 1622 | Tyrosine, Tryptophan | Proteins |
| 1658 | Amide I, C=C stretching | Nucleic acids |
| 1671 | Amide I vibration modeproteins, Cholesteol | Proteins/Lipids |
| 1680 | Amide I | Proteins |
| 1639 | Amide I | Proteins |
| 1737 | C-O stretching | Lipids |
| 1750 | C-O stretching | Lipids |

Table S3. Peak positions and assignments of major Raman spectral peaks observed in Leukemia cells in MCR

| The peak position/cm^-1^ | Biomolecules and vibrational assignments | Biological molecules |
| --- | --- | --- |
| ***Component 1*** |  |  |
| 621 | C–C twisting mode of phenylalanine | Proteins |
| 749 | Ring breathing of tryptophan | Proteins |
| 838 | Deformative vibrations of amine groups | Proteins |
| 938 | Proline, hydroxyproline, C-C skeletal of collagen backbone | Proteins |
| 1008 | C–H bending vibration of phenylalanine | Proteins |
| 1128 | C-N stretching (proteins) | Proteins |
| 1176 | C-H bending tyrosine (proteins) | Proteins |
| 1209 | Tretching of phenylalanine, Tyrosine, Tryptophan | Proteins |
| 1342 | CH3, CH2 wagging (collagen assignment) | Proteins |
| 1457 | CH2 stretching/CH3 asymmetric deformation | Proteins |
| 1661 | Amide I band | Proteins |
| ***Component 2*** |  |  |
| 719 | CN+(CH3) stretching | Lipid |
| 877 | C-C-N+syn stretching, C-O-C ring | Lipid |
| 1026 | Carbohydrates peak for solutions, Glycogen | Carbohydrates |
| 1087 | C-C skeletal of acyl backbone in lipid | Lipid |
| 1126 | C-C skeletal of acyl backbone in lipid  C-O,C-C, disaccharides, sucrose | Lipid  Carbohydrates |
| 1263 | Lipids | Lipid |
| 1301 | Triglycerides (fatty acids) | Lipid |
| 1343 | Glucose | Carbohydrates |
| 1445 | CH2 bending of lipids | Lipid |
| 1660 | ν(C=C) cis, lipids, fatty acids | Lipid |
| 1750 | C=O, ν(C=C) lipids, fatty acids | Lipid |
| ***Component 3*** |  |  |
| 672 | G (ring breathing modes in the DNA bases) | Nucleic Acids |
| 726 | A | Nucleic Acids |
| 785 | T; C; U; O-P-O stretching | Nucleic Acids |
| 828 | O–P–O asymmetric stretching of DNA | Nucleic Acids |
| 1093 | O-P-O symmetric stretching | Nucleic Acids |
| 1175 | C,G Cytosine, guanine | Nucleic Acids |
| 1241 | RNA | Nucleic Acids |
| 1373 | A; T; G | Nucleic Acids |
| 1443 | A; G | Nucleic Acids |
| 1485 | G; A; C-H def | Nucleic Acids |
| 1576 | G;A | Nucleic Acids |
| 1663 | DNA | Nucleic Acids |
| ***Component 4*** |  |  |
| 1220-1284 | A, T (ring breathing modes of the DNA/RNA bases)  Amide III (protein)  CH2 in plane deformation | Nucleic Acids  Lipid  Proteins |
| 1655-1680 | Lipid (C=C stretch)  Amide I (collagen assignment) | Lipid  Proteins |
